# A tale of two systems: practice patterns of a single group of emergency medical physicians in Taiwan and China

**DOI:** 10.1186/s12913-017-2606-4

**Published:** 2017-09-11

**Authors:** I-Anne Huang, Tang-Her Jaing, Chang-Teng Wu, Chee-Jen Chang, Shan-Hsuan Hsia, Nicole Huang

**Affiliations:** 1 Department of Pediatrics, Chang Gung Memorial Hospital, No. 222, Maijin Rd., Keelung, Taiwan; 2 Graduate Institute of Clinical Medical Sciences, College of Medicine, Chang Gung University, No.259, Wenhua 1st Rd., Guishan Dist, Taoyuan City, 333 Taiwan; 3 Institute of Public Health, National Yang Ming University, No.155, Sec. 2, Linong St., Beitou Dist, Taipei City, 112 Taiwan; 4 Department of Pediatrics, Chang Gung Memorial Hospital, No.123, Xiafei Rd., Haicang Dist, Xiamen City, China; 5 Department of Pediatrics, Chang Gung Children’s Hospital, 5. Fu-hsing St., Guishan Dist, Taoyuan City, 333 Taiwan; 6 Resources Center for Clinical Research, Chang Gung Memorial Hospital, 5. Fu-hsing St., Guishan Dist, Taoyuan City, 333 Taiwan; 7 Institute of Hospital and Healthcare Administration, National Yang Ming University, Room 101, Medical Building ll, No.155, Sec. 2, Linong St., Beitou Dist, Taipei City, 112 Taiwan

**Keywords:** Health system, Quality, Practice, Emergency, Children

## Abstract

**Background:**

The quality of pediatric emergency care has been a major concern in health care. Following a series of health system reforms in China, it is important to do this assessment of pediatric emergency care, and to explore potential influences of health care system. This study aimed to compare practice differences in treating children with respiratory illnesses in two emergency department (ED) settings within different health care systems: China and Taiwan.

**Methods:**

A pooled cross-sectional hospital-based study was conducted in two tertiary teaching hospitals in Xiamen, China and Keelung, Taiwan belong to the same hospital chain group. A team of 21 pediatricians rotated between the EDs of the two hospitals from 2009 to 2012. There were 109,705 ED encounters treated by the same team of pediatricians and 6596 visits were analyzed for common respiratory illnesses. Twelve quality measures in process and outcomes of asthma, bronchiolitis and croup were reported. Descriptive statistics and multiple logistic regression models were applied to assess. In order to demonstrate the robustness of our findings, we analyzed the data using an alternative modeling technique, multilevel modeling.

**Results:**

After adjustment, children with asthma presented to the ED in China had a significantly 76% lower likelihood to be prescribed a chest radiograph, and a 98% lower likelihood to be prescribed steroids and discharged home than those in Taiwan. Also, children with asthma presented to the ED in China had significantly 7.76 times higher risk to incur 24-72 h return visits. Furthermore, children with bronchiolitis in China (Odds ratio (OR): 0.21; 95% Confidence interval (CI): 0.17-0.28) were significantly less likely to be prescribed chest radiograph, but were significantly more likely to be prescribed antibiotics (OR: 2.19; 95% CI: 1.46-3.28).

**Conclusions:**

This study illustrated that although high quality care depends on better assessment of physician performance, the delivery of pediatric emergency care differed significantly between these two healthcare systems after holding the care providers the same and adjusting for important patient characteristics. The findings suggest that the features of the health care system may play a significant role.

## Background

Quality of pediatric emergency care has always been a major concern in health care [1–3]. Children constituted one-fifth of emergency department (ED) visits [4]. Variations in the quality of pediatric emergency care and their influencing factors are problems calling for more policy attention [5, 6]. Many factors could have influenced the delivery of pediatric emergency care [7–10]. In terms of individual characteristics, age, race, disease severity, socioeconomic status, and culture are important associating factors [10–12]. In addition, provider characteristics including demographic, training, specialty, patient volume, and adherence to clinical practice guidelines have also been identified as significant relating factors [13, 14]. More importantly, the influences of health system characteristics receive increasing attentions [5, 9, 15, 16]. Health system is described as complex adaptive systems by systems thinkers; it means free to respond to different stimuli in unpredictable ways and interconnected with the functions of other parts of a system [17, 18]. One intuitive approach is to observe the health care delivery in different health systems by controlling similar patients and care provider characteristics; China and Taiwan can serve as a useful and interesting comparison.

China has undergone a major health care reform since 2009. The characteristics of China’s health care reform are commonly described as “low level, wide coverage.” [19–22] The understanding of the differences in pediatric emergency care with other health systems has been relatively limited. Therefore, using Taiwan, a neighboring society with a well-known comprehensive insurance benefit coverage, as a comparison group may help to provide more insights about the delivery of pediatric emergency care under different health systems.

A large hospital chain group with the same information technology (IT) system and hospital operation in both China and Taiwan offers a unique opportunity to conduct this assessment [23]. Due to the shortage of pediatricians in China, a team of experienced emergency pediatricians from this hospital chain in Taiwan rotate their duties between the hospitals in Taiwan and in China. Therefore, the hospital-based data of this hospital chain during the period of a single team’s practice allow us to design a well-controlled pilot study to compare the practice patterns of pediatric emergency care between different health systems in China and Taiwan by maintaining the treating pediatricians and hospitals as a constant variable and then adjusting for comprehensive children’s characteristics.

### Aim of this study

Description of the differences between a same group of physicians in two emergency department settings within different systems served as an initial enquiry to establish lines of research into system differences’ impact on practitioner management and decision-making.

## Methods

### Study design and setting

The Chang Gung Memorial Hospital chain group has eight hospitals located in Taiwan and China. The hospital chain under study was first established in Taiwan in 1976 [23]. Two tertiary hospitals, one in Xiamen, China and the other in Keelung, Taiwan, were designated as the study settings. The Xiamen hospital has been in operation since 2008. The two hospitals were selected not only under the same IT and hospital operation systems but also the two cities were very similar in geographic characteristics, of the same language and of the same race, both are located in a suburb, and both overlook the sea, just across the Taiwan Strait, with subtropical climates in East Asia [24, 25]. The two hospitals were also the only tertiary care hospitals within 30 min travel time in these areas.

A group of pediatric emergency physicians regularly rotated their duty between the emergency departments of the two hospitals during the study period. All of the physicians are trained and board certified physicians in Taiwan. This study had a retrospective design that the physicians were not known the study measures during their practice. The team member staffing level and qualification, the admission wards, and the intensive care units were similar under a hospital chain’s operation. The triage classification was based on Taiwan Triage Acuity Scale, standardized in the same IT systems in both hospitals. The emergency pediatricians’ income is mainly fixed salary with a proportion of income related to patient volume, but not related to the volume and types of diagnostic testing, prescription, or procedures administered.

A pooled cross-sectional study design was conducted in two tertiary hospitals in Xiamen, China and Keelung, Taiwan to obtain a preliminary estimate of the difference in practice patterns between the same group of physicians in two emergency department settings within different health systems.

### Selection of participants

**Fig. 1:**
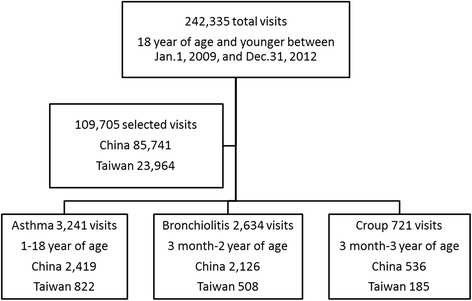
Study flow chart of sample selection

Figure 1 shows the selection process of our sample. There was a total of 242,335 pediatric emergency visits during the study period. Every physician included in this study had more than 120 patient visits in each hospital branch during the study period. Twenty-one physicians were enrolled in this study and each had managed 3000 to 5000 emergency visits. Only 109,705 ED pediatric visits treated by the same team of 21 physicians were identified. According to previous literatures [13, 14, 26, 27], three groups of pediatric visits were selected for analyses. The first group included children were aged from one to 18 years with a primary or secondary diagnosis of asthma (N1 = 3241). The second group included children aged from 3 months to 2 years with a primary or secondary diagnosis of bronchiolitis (N2 = 2634), and the third group included children aged between 3 months to 3 years with a primary or secondary diagnosis of croup (N3 = 721).

### Health systems

The quantity of practitioners in a geographic area was four and five physicians per square kilometers in Keelung and Xiamen, respectively [24, 25, 28–30]. There were both 1.9 physicians per thousand people in Keelung and Xiamen [28–30]. The financing is different in the two health systems. Children that visited the ED in Xiamen may or may not have insurance coverage [31, 32]. Even with the insurance, people need to pay a large proportion of ED expenses out-of-pocket [32]. The patient’s cost sharing was reduced from 79% in 2009 to 65% in 2012 [32]. However, in Keelung, Taiwan’s National Health Insurance is mandatory and offers comprehensive coverage for emergency services [33]. No co-payment or very minimal co-payment is levied for children’s emergency care [33]. Despite the differences between the two health systems, they share one feature in common. People can seek care from any provider they like and their access to specialists is not limited or managed by a gatekeeper.

### Measures

As respiratory tract infection is a main reason of pediatric emergency visits, many quality indicators established for pediatric emergency care are specific to pediatric respiratory illnesses, including asthma, bronchiolitis, and croup [13, 14, 27, 34, 35]. Diagnoses were coded using the International Classification of Diseases, Ninth Revision, Clinical Modification (ICD-9-CM) classification in Taiwan, and the International Classification of Disease, Tenth Revision (ICD-10) codes in China. Table 1 lists the ICD-9-CM in Taiwan and ICD-10 codes in China for these diagnoses.

**Table 1:** ICD-9-CM and ICD-10 Codes Used for the Definition of Patient Population

	Asthma	Bronchiolitis	Croup
ICD-9-CM	493.0	466	464
	493.01	466.0	464.0
	493.02	466.1	464.2
	493.1	466.11	464.20
	493.10-12	466.19	464.21
	493.9	487.1	464.4
	493.90-92		
ICD-10	J45	J21	J04
	J450	J210	J040
	J45001-05	J21051	J04001
	J45051-53	J218	J04002
	J451	J21851	J04004
	J45101	J219	J04006
	J45151	J21901-03	J042
	J45152	J10	J04201
	J458	J101	J05
	J45851	J108	J050
	J459	J11	J05001
	J45901-03	J111	J05051
	J45951	J11101	J06
	J45952	J118	J06001
	J46		J06002
	J46X01		J06051
	J46X51		

The hospital ED records of the selected pediatric visits between 2009 and 2012 were reviewed in these two tertiary hospitals. Patient-level data included demographics such as age, gender, triage status, season, the timing of the visit, and the insurance status; the clinical information of diagnoses, procedures with imaging, medication, and return visits information were collected from hospital electronic medical records. The standardized processes for asthma, bronchiolitis and croup were the same in the two hospitals during the study period. The protocol for the conduct of this study was reviewed and approved by the Chang Gung Memorial Hospital Institutional Review Board.

According to previous literature, different sets of quality indicators were used for asthma, bronchiolitis, and croup [13, 14, 26, 27, 35]. Four process measures (chest radiograph, the prescription of antibiotics, and steroids in those discharged or admitted) and two outcomes measures (unplanned return visits within 24 h and 24-72 h) were used for asthma visits of children aged between one to 18 years. Two process measures (chest radiograph and antibiotics prescription) were used for bronchiolitis visits of children aged 3 months to 2 years. Three process measures (chest or lateral neck radiograph, the prescription of steroids, and the patient admitted in ED not receiving steroids) and one outcome measure (unplanned return visit within 24 h) were used for croup visits of children aged between 3 months and 3 years.

### Statistical analysis

Descriptive statistics were presented. Chi-square tests and t-tests were used to compare patient characteristics between the Xiamen and Keelung hospitals. Univariate and multiple logistic regression models were applied to assess the quality of pediatric emergency care in 12 quality indicators selected in Xiamen while using the Keelung hospital data as a reference. Basically, we selected significantly related factors which have been identified in the existing literature to construct our final multiple logistic regression models [13, 14]. The comparisons were adjusted for age, gender, triage, season, and time of the visit with 95% confidence interval for asthma and bronchiolitis groups. For croup, the comparison was adjusted for gender, triage, season, and time of the visit. Since we limited our selection of croup visits to children aged between 3 months to 3 years, the age range was somewhat narrow and within some age categories, the number of children was very small and did not allow for a meaningful comparison.

Furthermore, in the main analyses, triage was reconstructed as a dichotomous variable: triage level 1-3 (resuscitation, emergent, urgent) and triage level 4-5 (semi-urgent, non-urgent). In order to examine whether the results remained robust among more urgent and severe visits, we conducted sensitivity analyses by limiting our analyses to the visits with a triage level 1-3 (resuscitation, emergent, urgent). In order to reduce potential biases from clinical variations among the visits, we first limited our analyses among riskier patients with a triage level 1-3. In addition, we conducted another sensitivity analyses to limit the visits with a primary diagnosis of asthma, bronchiolitis and croup. Also, in order to demonstrate the robustness of our findings, we analyzed the data using an alternative modeling technique, multilevel modeling. Due to a possible nested structure of the data (visits nested within providers, we constructed two-level models using PROC GLIMMIX. The first level was visits and the second level was providers. Within the Xiamen sample, quality measures were compared between children both with and without insurance. The level of statistical significance of 0.05 was chosen. All statistical analyses were performed using the statistical software SAS v9.3 (SAS Institute, Inc., Cary, NC).

## Results

Tables 2, 3 and 4 presents distributions of patient characteristics between Xiamen, China and Keelung, Taiwan. Only 44.3%, 19.7%, 24.4% of children in the asthma, bronchiolitis, and croup visits in Xiamen had an insurance coverage. More importantly, whereas more than 85% of pediatric ED visits for asthma (88.0%), bronchiolitis (89.8%), or croup (95.1%) in Keelung were having triage at levels 1-3, less than 50% of pediatric ED visits for these conditions (asthma: 18.6%; bronchiolitis: 29.5%; croup: 44.6%) in Xiamen were having triage at levels 1-3. The percentage of these ED visits resulting in admission in Keelung was 3-4 times higher than that in Xiamen. A significant difference in the timing of ED visits was also observed. More than 50% of ED visits in Xiamen for these three conditions were made in daytime shifts, but less than 32% of pediatric ED visits for these conditions in Keelung occurred during daytime shifts.

**Table 2:** Sample Characteristics of Pediatric Emergency Department Visits for Asthma by Two Health Systems

Astma, 1-18 yr	Overall *N* = 3241	China *N* = 2419	Taiwan *N* = 822	*P* value
Age, mean ± SD, yr	4.7 ± 2.9	4.0 ± 2.3	6.9 ± 3.5	<0.001
Gender, total n (%)				0.053
Male	2134 (65.8%)	1570 (64.9%)	564 (68.6%)	
Female	1107 (34.2%)	849 (35.1%)	258 (31.4%)	
Insurance, total n (%)				<0.001
Yes	1893 (58.4%)	1071 (44.3%)	822 (100.0%)	
No	1348 (41.6%)	1348 (55.7%)	0 (0.0%)	
Triage level, total n (%)				<0.001
1-3: resuscitation, emergent, urgent	1171 (36.1%)	448 (18.5%)	723 (88.0%)	
4-5: semiurgent, nonurgent	2070 (63.9%)	1971 (81.5%)	99 (12.0%)	
Season, total n (%)				0.772
Spring, Summer	1394 (43.0%)	1044 (43.2%)	350 (42.6%)	
Fall, Winter	1847 (57.0%)	1375 (56.8%)	472 (56.4%)	
Time of visit, total n (%)				<0.001
Day	1711 (52.8%)	1498 (61.9%)	213 (25.9%)	
Night	1530 (47.2%)	921 (38.1%)	609 (74.1%)	
Disposition, total n (%)				<0.001
Discharged	3101 (95.7%)	2360 (97.6%)	741 (90.2%)	
Admitted	140 (4.3%)	59 (2.4%)	81 (9.9%)

**Table 3:** Sample Characteristics of Pediatric Emergency Department Visits for Bronchiolitis by Two Health Systems

Bronchiolitis, 3 m-2y	Overall *N* = 2634	China *N* = 2126	Taiwan *N* = 508	*P* value
Age, mean ± SD, yr	1.0 ± 0.6	1.0 ± 0.6	1.2 ± 0.6	<0.001
Gender, total n (%)				0.002
Male	1694 (64.3%)	1397 (65.7%)	297 (58.5%)	
Female	940 (35.7%)	729 (34.3%)	211 (41.5%)	
Insurance, total n (%)				<0.001
Yes	926 (35.2%)	418 (19.7%)	508 (100.0%)	
No	1708 (64.8%)	1708 (80.3%)	0 (0.0%)	
Triage level, total n (%)				<0.001
1-3: resuscitation, emergent, urgent	1083 (41.1%)	627 (29.5%)	456 (89.8%)	
4-5: semiurgent, nonurgent	1551 (58.9%)	1499 (70.5%)	52 (10.2%)	
Season, total n (%)				0.006
Spring, Summer	1400 (53.2%)	1158 (54.5%)	242 (47.6%)	
Fall, Winter	1234 (46.8%)	968 (45.5%)	266 (52.4%)	
Time of visit, total n (%)				<0.001
Day	1638 (62.2%)	1477 (69.5%)	161 (31.7%)	
Night	996 (37.8%)	649 (30.5%)	347 (68.3%)	
Disposition, total n (%)				<0.001
Discharged	2351 (89.3%)	1970 (92.7%)	381 (75%)	
Admitted	283 (10.7%)	156 (7.3%)	127 (25%)

**Table 4:** Sample Characteristics of Pediatric Emergency Department Visits for Croup by Two Health Systems

Croup, 3 m-3y	Overall *N* = 721	China *N* = 536	Taiwan *N* = 185	*P* value
Age, mean ± SD, yr	1.5 ± 0.8	1.5 ± 0.8	1.6 ± 0.8	0.055
Gender, total n (%)				0.646
Male	493 (68.4%)	364 (67.9%)	129 (69.7%)	
Female	228 (31.6%)	172 (32.1%)	56 (30.3%)	
Insurance, total n (%)				<0.001
Yes	316 (43.8%)	131 (24.4%)	185 (100.0%)	
No	405 (56.2%)	405 (75.6%)	0 (0.0%)	
Triage level, total n (%)				<0.001
1-3: resuscitation, emergent, urgent	415 (57.6%)	239 (44.6%)	176 (95.1%)	
4-5: semiurgent, nonurgent	306 (42.4%)	297 (55.4%)	9 (4.9%)	
Season, total n (%)				0.018
Spring, Summer	412 (57.1%)	320 (59.7%)	92 (49.7%)	
Fall, Winter	309 (42.9%)	216 (40.3%)	93 (50.3%)	
Time of visit, total n (%)				<0.001
Day	324 (44.9%)	269 (50.2%)	55 (29.7%)	
Night	397 (55.1%)	267 (49.8%)	130 (70.3%)	
Disposition, total n (%)				<0.001
Discharged	640 (88.8%)	501 (93.5%)	139 (75.1%)	
Admitted	81 (11.2%)	35 (6.5%)	46 (24.9%)	

Table 5 compares the disease-specific quality measures of asthma, bronchiolitis and croup between Xiamen, China and Keelung, Taiwan. In terms of the process indicators for asthma care, while there was only a slight difference in the antibiotic prescription rate between the two settings, the prescription rates of chest radiograph (5.7%) and steroids (2.0% at ED discharge) were substantially lower in Xiamen, China than in those in Keelung, Taiwan (chest radiograph: 26.2%; steroids at ED discharge: 54.1%). There was only a slight difference in the admitted asthma patients with steroid prescription rates at the ED between the two settings (Xiamen, China: 53.4%; Keelung, Taiwan: 47.9%). After adjustment, children with asthma presented to the ED in Xiamen had a significantly 76% lower likelihood to be prescribed a chest radiograph, and a 98% lower likelihood to be prescribed steroids and discharged home, but no significant difference was observed for either antibiotic prescription or steroids at admission. In terms of outcome measurements, after adjustment, although there was no statistically significant difference in return visit within 24 h, children with asthma presented to the ED in Xiamen had a significantly 7.76 times higher risk to return to the hospital between 24 and 72 h when compared to those in the Keelung hospital.

**Table 5:** Comparative Data between China and Taiwan in Quality Indicators

Asthma, 1-18y, total n (%)	China *N* = 2419	Taiwan *N* = 822	Unadjusted Odds Ratio (95% CI) China vs Taiwan	*P* value	Adjusted^a^ Odds Ratio (95% CI) China vs Taiwan	*P* value
Radiographs	137 (5.7%)	215 (26.2%)	0.17 (0.13-0.21)	<0.001	0.24 (0.17-0.33)	<0.001
Antibiotics	144 (6.0%)	60 (7.3%)	0.80 (0.59-1.10)	0.170	0.99 (0.65-1.52)	0.966
Steroids and discharged home^b^	47 (2.0%)	401 (54.1%)	0.02 (0.02-0.03)	<0.001	0.02 (0.01-0.03)	<0.001
Admitted with steroid in the ED^c^	31 (53.4%)	39 (47.9%)	0.26 (0.16-0.42)	<0.001	0.45 (0.23-0.86)	0.017
Unplanned return visit within 24 h	30 (1.2%)	23 (2.8%)	0.44 (0.25-0.76)	0.002	0.61 (0.28-1.31)	0.205
Unplanned return visit between 24 and 72 h	48 (2.0%)	2 (0.2%)	8.30 (2.27-70.63)	0.001	7.76 (1.62-37.17)	0.010
Bronchiolitis, 3 m-2y, total n (%)	China *N* = 2126	Taiwan *N* = 508				
Radiographs	304 (14.3%)	267 (52.6%)	0.15 (0.12-0.19)	<0.001	0.21 (0.17-0.28)	<0.001
Antibiotics	223 (10.5%)	37 (7.3%)	1.49 (1.04-2.14)	0.030	2.19 (1.46-3.28)	<0.001
Croup, 3 m-3y, total n (%)	China N = 536	Taiwan N = 185				
Radiographs	85 (15.9%)	112 (60.5%)	0.12 (0.08-0.18)	<0.001	0.12 (0.08-0.19)	<0.001
Steroids	282 (52.6%)	118 (63.8%)	0.63 (0.45-0.89)	0.008	0.93 (0.63-1.38)	0.713
Admitted without steroids in ED^c^	14 (40.0%)	21 (45.7%)	0.79 (0.33-1.94)	0.611	0.87 (0.33-2.30)	0.774
Unplanned return visit within 24 h	17 (3.2%)	7 (3.8%)	0.83 (0.34-2.04)	0.689	0.59 (0.20-1.74)	0.338

Taiwan serves as the reference group

^a^Adjust for age, gender(reference = female), triage(reference = triage level 4-5), season(reference = spring & summer) and time of visit(reference = night) in Asthma & Bronchiolitis

^a^Adjust for gender(reference = female), triage(reference = triage level 4-5), season(reference = spring & summer) and time of visit(reference = night) in Croup

^b^Percentage with the discharge as the denominator

^c^Percentage with the admitted as the denominator

Children with bronchiolitis in Xiamen (OR: 0.21; 95% CI: 0.17-0.28) were significantly less likely to be prescribed chest radiograph than those in Keelung, but were significantly more likely to be prescribed antibiotics (OR: 2.19; 95% CI: 1.46-3.28). In terms of croup care, children in Xiamen were significantly less likely to be prescribed radiographs (OR: 0.12; 95% CI: 0.08-0.19) and to be admitted (OR: 0.27; 95% CI: 0.15-0.46) than children in Keelung. In terms of outcome indicators, no significant difference was observed in the return visit within 24 h between children with croup in two settings.

Furthermore, we also compared 12 quality measures between pediatric ED visits both with and without health insurance in pediatric emergency visits in Xiamen (Table 6). There was no significant difference in quality of ED care observed between children with and without insurance. Finally, as for sensitivity analyses, the results of adopting multilevel models yielded similar significant findings. The results of all other sensitivity anlayses also remained robust.

**Table 6:** Comparative Data between China with Insurance or not in Quality Indicators

Asthma, 1-18y, total n (%)	China no insurance *N* = 1384	China with insurance *N* = 1071	Unadjusted Odds Ratio (95% CI) With vs No insurance	*P* value	Adjusted^a^ Odds Ratio (95% CI) With vs No insurance	*P* value
Radiographs	81 (6.0%)	56 (5.2%)	0.86 (0.61-1.23)	0.410	0.86 (0.60-1.23)	0.410
Antibiotics	80 (5.9%)	64 (6.0%)	1.01 (0.72-1.41)	0.966	1.05 (0.74-1.48)	0.795
Steroids and discharged home^b^	24 (1.8%)	23 (2.2%)	1.21 (0.68-2.16)	0.516	1.25 (0.69-2.25)	0.465
Admitted with steroid in the ED^c^	17 (47.2%)	14 (60.9%)	1.04 (0.51-2.11)	0.920	1.90 (0.58-6.17)	0.288
Unplanned return visit within 24 h	15 (1.1%)	15 (1.4%)	1.26 (0.61-2.59)	0.525	1.25 (0.60-2.60)	0.554
Unplanned return visit between 24 and 72 h	23 (1.7%)	25 (2.3%)	1.38 (0.78-2.44)	0.271	1.34 (0.75-2.39)	0.331
Bronchiolitis, 3 m-2y, total n (%)	No insurance *N* = 1708	With insurance *N* = 418				
Radiographs	245 (14.3%)	59 (14.1%)	0.98 (0.72-1.33)	0.904	1.11 (0.81-1.53)	0.514
Antibiotics	189 (11.1%)	34 (8.1%)	0.71 (0.49-1.04)	0.080	0.71 (0.48-1.05)	0.089
Croup, 3 m-3y, total n (%)	No insurance *N* = 405	With insurance *N* = 131				
Radiographs	68 (16.8%)	17 (13.0%)	0.74 (0.42-1.31)	0.299	0.75 (0.42-1.33)	0.317
Steroids	222 (54.8%)	60 (45.8%)	0.70 (0.47-1.03)	0.073	0.73 (0.48-1.09)	0.123
Admitted without steroids in ED^c^	12 (40.0%)	2 (40.0%)	0.51 (0.05-2.33)	0.534	1.04 (0.10-10.43)	0.974
Unplanned return visit within 24 h	13 (3.2%)	4 (3.1%)	0.95 (0.30-2.96)	1	0.93 (0.30-2.92)	0.900

No insurance in China serves as the reference group

^a^Adjust for age, gender(reference = female), triage(reference = triage level 4-5), season(reference = spring & summer) and time of visit(reference = night) in Asthma & Bronchiolitis

^a^Adjust for gender(reference = female), triage(reference = triage level 4-5), season(reference = spring & summer) and time of visit(reference = night) in Croup

^b^Percentage with the discharge as the denominator

^c^Percentage with the admitted as the denominator

## Discussion

This study represents a compelling and fascinating glimpse into two pediatric emergency care delivery influenced by different healthcare systems. Although in both Keelung, Taiwan and Xiamen, China, social health care insurance programs are available, the programs are difference in eligibility criteria, extent of benefit coverage, cost-sharing obligations, delivery care system, and reimbursement approaches. Although high quality care depends on better assessment of physician performance, the delivery of pediatric emergency care differed significantly between these two healthcare systems after holding the care providers the same and adjusting for important patient characteristics. Compared to pediatric patients in Keelung, pediatric patients in Xiamen were significantly less likely to receive more expensive diagnostic procedures and medications (i.e. steroid prescription for asthma when discharged home), but more likely to receive potentially inappropriate antibiotics for bronchiolitis, and to incur ER return visits for asthma. Similar potentially inappropriate patterns were also observed for children with insurance in Xiamen.

The pattern of pediatric emergency care for asthma, croup, and bronchiolitis observed in Xiamen, China may be attributable to insufficient health insurance coverage [21, 22, 32, 36], different culture/health beliefs [37–39], and lack of pediatric access to primary care providers, which may worth further investigations. Firstly, unlike the full insurance coverage of emergency care offered to children in Taiwan, on average, a high cost-sharing (i.e. 79% in 2009 and 65% in 2012) was associated with emergency services for children in Xiamen, China. According to the existing literature, the prescription of chest radiograph in asthma should be decreased in use and steroids at discharge should be given; the extremely low rates in Xiamen, China were noted [13, 14, 26, 27, 35]. Substantially lower utilization of the guideline recommended, but relatively expensive diagnostic imaging services may suggest the existence of financial barriers. After limiting the samples to children with the triage level with resuscitation, emergent, and urgent visits, the results remained the same.

One plausible explanation is that only 44.3%, 19.7%, and 24.4% of the children, who had made ER visits for asthma (one - 18 years), bronchiolitis (3 months - 2 years), and croup (3 months- 3 years), respectively, in Xiamen had health insurance coverage. The lack of sufficient insurance poses substantial financial barriers for children to receive quality emergency care. The high percentage of children visiting ER without insurance raises a serious concern. Children without insurance in Xiamen were mostly likely be from migrant families and temporary residents with low socioeconomic status [22, 36]. More specifically, even when the children had health insurance in Xiamen, the insurance coverage may not be sufficient in relative low rates of diagnostic tests.

Another interesting finding is that although steroids are recommended for asthma children when discharged home and it is relatively inexpensive in Xiamen, China, a significantly lower likelihood of steroid prescription for asthma when discharged home was observed in Xiamen. According to empirical clinical evidences [27, 38], corticosteroids has been proven effective in controlling symptoms for children with chronic asthma and managing acute onset of pediatric asthma. Appropriate use and application of corticosteroids in front-line pediatric emergency care can help to reduce hospital and emergency room use. Different patterns for prescribing steroids between Xiamen and Keelung may indicate potential differences in culture and people’s attitudes toward specific medications in Taiwan and China. Previous studies revealed national and racial disparities in the patient’s knowledge about asthma and its management [11, 12, 37–39]. South Asia parents tended to believe that inhaled corticosteroids did more harm than good when compared to the beliefs of Caucasian parents (OR 3.19, CI 1.22-8.34) [37]. One survey investigated parents of uncontrolled asthma children and found that more parents favored short-acting beta-agonist than inhaled corticosteroids in China (70%) than in Taiwan (35%) [38]. Another study in China also found that parent’s fears and concerns about the side effects of steroids was one strongest reason why there was non-adherence to medication among children with asthma [39]. On the other hand, the prescription of antibiotics in bronchiolitis were more commonly prescribed in Xiamen, China than Keelung, Taiwan. To reduce potentially inappropriate prescribing, further qualitative investigations for a deeper understanding the factors driving prescribing behaviors is essential. Parent’s beliefs and knowledge shall not be overlooked and may have also influence the health care delivery in the health systems and then change the quality of emergency care received by children.

Thirdly, compared to the ER utilization patterns in Keelung, Taiwan, the utilization of pediatric emergency care in Xiamen had more day-shift visits, more 72-h returns, and more visits with lower triage severity. Whereas community primary care physicians are mostly general practitioners in Xiamen, the findings led to concerns about the availability of community medical resources or accessibility to pediatricians for children in Xiamen. As many of these visits could be appropriately managed by primary care physicians or pediatricians at community settings, it is important to further investigate factors driving parents to seek emergency care for less urgent conditions of their children [40].

A few limitations are notable. First, because our study was hospital-based, performance measurements may be underestimated if the children visited other hospitals for the same episode; we were not able to obtain their utilization of health services in other hospitals. Second, residual confounding may be plausible since information such as parental knowledge, beliefs, or income are not available in our dataset. Third, the study only assessed the quality of three commonly treated respiratory conditions in ER. The results may not be generalizable to other pediatric emergency conditions. Fourth, since our data were limited to one hospital chain group, the results may not be generalizable to other hospitals in China. However, using the detailed information from the hospital information system and the unique staffing rotation feature of this hospital chain allowed us to better control for care providers and patient characteristics. Furthermore, healthcare access and insurance coverage in Xiamen, in general, is better than most of the other cities and regions in China, and so treatment patterns and quality of pediatric care between China and Taiwan may be larger than we observed in our study.

## Conclusions

In summary, this was the first study to illustrate the differences in pediatric emergency care delivery in China provided by a single team’s practice under two different health care systems using Taiwan as a comparison group. Compared to the situation in Taiwan, serious financial barriers to appropriate diagnostic and treatment procedures still exist among the pediatric population in China, which poses a threat to quality of pediatric emergency care delivery. In addition, parental negative attitudes toward steroids in asthma control and inadequate pediatric resources at community settings may have also influenced the utilization and quality of emergency care in China. Our findings suggest that many pediatric patients may have used ED as a usual source of care for non-urgent conditions. That implies barriers to access for appropriate children’s care. The findings may serve as references to future quality improvement efforts in pediatric emergency care in China and Taiwan.

## Abbreviations

CI: Confidence interval
ED: Emergency department
ICD-10: International Classification of Disease, Tenth Revision
ICD-9-CM: International Classification of Diseases, Ninth Revision, Clinical Modification
IT: Information technology
N: Numbers
OR: Odds ratio
